# Co-existence of bla_IMP_, bla_NDM-1_, and bla_SHV_, genes of Pseudomonas aeruginosa isolated from Quetta: Antimicrobial resistance and clinical significance

**DOI:** 10.12669/pjms.39.5.7188

**Published:** 2023

**Authors:** Mohammad Din, Mohammad Arif Awan, Sadeeq ur Rahman, Mohammad Ali, Momina Aslam

**Affiliations:** 1 Mohammad Din, PhD Department of Pathology, Bolan Medical College/Complex Hospital Quetta, Baluchistan, Pakistan; 2 Mohammad Arif Awan, PhD CASVAB, University of Baluchistan, Quetta, Pakistan; 3 Sadeeq-ur-Rahman, PhD Department of Microbiology, College of Veterinary, Sciences and Animal Husbandry, Abdul Wali Khan University, Mardan, Khyber-Pakhtunkhwa, Pakistan; 4 Mohammad Ali, PhD CASVAB, University of Baluchistan, Quetta, Pakistan; 5 Momina Aslam, M.Phil Department of Pathology, Bolan Medical Complex Hospital Quetta, Baluchistan, Pakistan

**Keywords:** Carbapenemase, XDR, blaSHV, blaIMP, blaNDM-1, Quetta

## Abstract

**Objective:**

Molecular detection and co-presence of carbapenem-resistant genes in the isolates of *Pseudomonas aeruginosa* are less commonly reported from Quetta. In the present study, we determined to highlight the antibiotic sensitivity profile and genetic mechanism of carbapenem resistance.

**Methods:**

The cross-sectional study was conducted from May to September 2018 at the Hi-tech laboratory, Centre for Advance Studies in Vaccinology and Biotechnology, University of Baluchistan, Quetta. Biochemical and molecular methods were ascertained for the recognition of the isolates and minimum inhibitory concentration was performed using E-test and broth microdilution methods. The molecular basis of carbapenemase activity was determined by identifying carbapenemase genes in the isolates.

**Results:**

Of the (n=23) *P. aeruginosa* isolated from pus aspirates obtained from surgical/burn units, we have detected bla_IMP_ (n=7/8) 87.5%, bla_NDM-1_ (n=5/8) 62.5%, and bla_SHV_ (n=4/8) 50%. The co-existence of multiple antibiotic-resistant genes, bla_IMP_, bla_NDM-1_ and bla_SHV_ was found in (n=2/8) 25% isolates. These isolates displayed resistance against a range of antimicrobials from β-lactams, tetracyclines, cephalosporins, quinolones, monobactams, aminoglycosides, sulphonamides, phosphoric acid, macrolides, and polypeptide groups, suggesting extensive-drug resistance.

**Conclusion:**

The emergence of MBL and ESBL producers is an alarming threat in the region. It is of great importance to determine the resistance mechanism of bacterial bugs. The lack of new antimicrobials particularly against gram-negative bacteria is quite alarming worldwide.

## INTRODUCTION

*Pseudomonas aeruginosa* is an obligate aerobic, saprophytic, non-fermenting, gram-negative bacillus mostly inhabits in humid environment.^1^ In the recent previous year 2017, the WHO declared 12 bacterial isolates that were the greatest threat to human health, amongst those carbapenem-resistant *P. aeruginosa* was given critical priority.^2^ The discovery of antimicrobials was thought a symbol of hope in human fight against infections, but in the developing countries it is still the foremost cause of death. The emergence of resistance is the leading barrier in treating infectious diseases.^3^

Antimicrobial susceptibilities are classified by the European Centre for Disease Control (ECDC) and the Centers for Disease Control and prevention (CDC), MDR as non-susceptible to at least one antimicrobial agent in three or more groups, XDR as non-susceptible to at least one antimicrobial agent in all but two or fewer classes (i.e., bacterial isolates remain sensitive to only one or two groups), and PDR as non-susceptible to all antibacterial classes.^4^ Carbapenem antimicrobials are usually considered to be the last-line agents to treat severe infections caused by *P. aeruginosa*. Though, the recent rise in the occurrence of carbapenem-resistant *P. aeruginosa* hospital acquired isolates is of great concern.^5^

There are various techniques reported to determine the MICs for both ESBL positive and ESBL negative pathogenic bacteria that included Vitek-2, micro-scan, sensititre, agar dilution, broth microdilution, broth macrodilution, phoenix automated system, and E-test, that also help in the evaluation of different therapeutic drugs.^6^ Therefore, the molecular-based confirmatory test also has a dynamic role in the detection of ESBLs. It showed a high level of specificity as well as sensitivity in the identification of specific ESBL derivatives found in clinical species.

With the prevalence of carbapenem resistant genes and their rapid spreading due to mobile genetic elements, these antimicrobials are restricted. Different variants of ESBLs and MBLs such as bla_IMP_, bla_NDM_, bla_VIM_, and bla_SHV_ producing *P. aeruginosa* have been isolated from different regions of the world.^7^ Therefore, an extensive survey is necessary to further explore the carbapenemase-producing *P. aeruginosa* globally. The aim of this research investigation was to identify the distribution of ESBLs and MBLs (carbapenemase) genes of Ambler class amongst the clinical isolates of *P. aeruginosa* in this region. As *P. aeruginosa* is the leading nosocomial superbug in the hospital settings and source of greater resistance. Moreover, partial work has been done in the province of Baluchistan on this globally emerging threat. Therefore, the present study was planned which would give guidelines to the physicians to treat the patients in a proper way and anticipated to recommend the patients antibiotics after going through culture and sensitivity testing, thus avoiding the consequences of worse resistance.

## METHODS

Three hundred and fifty (n=350) clinical samples (pus aspirates) were taken aseptically from different units of tertiary care hospitals. Patients of both gender and all age groups were included in the present study. All the samples were obtained aseptically in sterile 5cc syringes (Becton Dickinson, USA) and with sterilized cotton swabs (autoclaved at 121°C at 15 psi for 20 minutes) from surgical wards, outdoor patient departments (OPDs), burns wards, OPD and burns intensive care units (ICUs).

### Ethical Approval:

The bioethical approval for the present study was taken from the Institutional Bioethical Committee Bolan Medical Complex Hospital, No. Estt-DA-11 BMCH-AP-1587-8 Quetta, Baluchistan.

Patients with oozing pus from their wounds after several antibiotic therapies were enrolled in the present study. Complete history was taken from the patients about time and duration of infection, pre-existing clinical complications, antibiotic therapy, and area/climate where they were living.

Selection of the patients with antimicrobial-resistant infections was based on complete clinical history as well as several previous antibiotic therapies. All the patients were advised to stop antibiotic therapy for 72 hours before sample collection because, samples contain more bacterial load prior to the antibiotic therapy.^8^ Samples were labeled and transported to the microbiology laboratory immediately.

Conventional microbiological methods were applied for bacterial identification. All the samples were streaked simultaneously on MacConkey and Cetrimide agar plates (Oxoid, UK) and were incubated aerobically for 24 hours at 37°C.^9^ The isolates of *P. aeruginosa* were identified by the analytical profile index (API20NE) system (bioMerieux, France) according to the manufacturer’s instructions.^10^

Standardized antibiotic sensitivity test, disc diffusion Kirby Bauer method, and 0.5 McFarland turbidity standard were performed against all the isolates of *P. aeruginosa*. The minimum inhibitory concentrations (MICs) of extensively drug-resistant *P. aeruginosa* isolates were achieved by commercially available E-test strips (Oxoid, UK and Liofilchem, Italy) and broth microdilution method as previously described.^11^ The CLSI and FDA breakpoints were followed in the result interpretation. The broth-microdilution method was used to determine the MICs of colistin and polymyxin-B in cation-adjusted Mueller Hinton broth (CAMHB), following the CLSI guidelines^12^, using polymyxin B and colistin sulfate powders (Sigma-Aldrich, Germany). The broth microdilution panels were incubated at 35°C for 16-20 hours and the results were interpreted as described.^13^

The gram-negative isolates of *P. aeruginosa* showing zone of inhibition ≤25mm for ceftriaxone and/or ≤22mm for ceftazidime and/or ≤27mm for cefotaxime were screened for potential ESBL production following CLSI guidelines.^12^ The isolates of *P. aeruginosa* were initially screened phenotypically by double disk diffusion synergy method for ESBLs detection.^14^ This new phenotypic test was performed to detect the carbapenemase activity in *P. aeruginosa* isolates by inactivating the carbapenem. The mCIM is a very healthy and gainful phenotypic method.^12^

The genomic DNA of extensive drug-resistant isolates of *P. aeruginosa* was extracted by a commercially available kit (WizPrep, Korea, lot No. 4A0619-07) following the manufacturer’s directions. The specie-specific primers were used to identify the 16S rRNA gene in the isolates of *P. aeruginosa*, using the following set of primers, PA-SS- F- GGGGGATCTTCGGACCTCA and PA-SS-R CCTTAGAGTGCCCACCCG.^15^ The gel was visualized in the gel documentation system (wealtec dolphin-view S # WDV-50710004 USA) for the amplicon size of 956 bp. The plasmid DNA of extensive drug-resistant isolates of *P. aeruginosa* was extracted by a commercially available plasmid extraction kit (BioLabs, UK) following the manufacturer’s directions.

All the ESBL and MBL (carbapenemase) positive isolates of *P. aeruginosa* (n=23) based on phenotypic detection were further confirmed by singleplex PCR for the detection of bla_NDM-1_, bla_IMP_, and bla_SHV_ using the following sets of primers (Table-I).

**Table-I:** Primer sequences used for PCR of drug resistance genes in ESBL & MBL (carbapenemase) producers.

Gene	Oligonucleotide Sequences	Amplicon Size (bp)	References
bla_NDM-1_	F-5’-GGGCAGTCGCTTCCAACGGT-3’	475	(16)
	R-5’-GTAGTGCTCAGTGTCGGCAT-3’		
bla_IMP_	F-5’-TGAAAGGCTTATCTGTATTC-3’	740	(17)
	R-5’- TAGTTGCTTGGTTTTGATG-3’		
bla_SHV_	F-5’-AGCCGCTTGAGCAAATTAAAC-3’	713	(18)
	R-5’- ATCCCGCAGATAAATCACCAC-3’		

The sequencing of the PCR product of specie-specific 16S rRNA, bla_IMP_, bla_NDM-1_, and bla_SHV_ genes in the representative isolates of *P. aeruginosa* was performed commercially with DNA sequencer using forward primers.

## RESULTS

**Fig.1:**
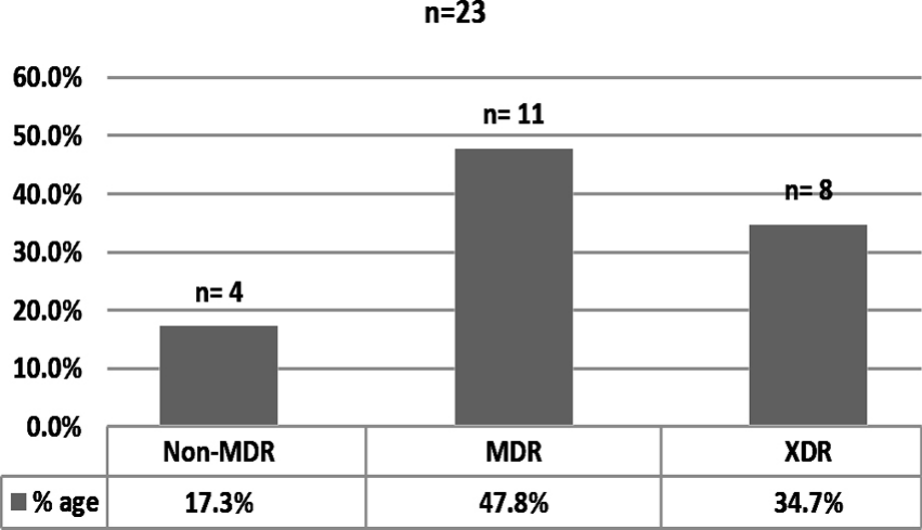
Number & percentage of non-MDR, MDR, & XDR *P. aeruginosa*.

Based on disc diffusion Kirby Bauer method and 0.5 McFarland turbidity standard (data not shown), out of (n=23) *P. aeruginosa* isolates (n=4) 17.4% were found as non MDR, (n=11) 47.8% MDR, and (n=8) 34.7% XDR (Fig.1). These isolates were resistant against anti-pseudomonal antibiotics belonging to carbapenems, beta-lactam, aminoglycosides, monobactams, fluroquinolones, penicillins, cephalosporins, and lipopeptides groups. The susceptibility patterns such as, non-MDR, MDR, and XDR were categorized as terminologies created by the CDC, and ECDC.^4^

The MICs data and percentage of XDR isolates of *P. aeruginosa* against each tested antibiotics has shown in (Table-II & III). Out of isolates (n=23), eight were categorized XDR, as they were only sensitive to one or two families of antimicrobials tested. The results of MICs were compared with disc diffusion method, in disc diffusion susceptibility percentage of tigecycline was S= 12.5%, I=50% and R=37.5% whereas, MICs showed S= 25%, I=12.5% and R=62.5%. The difference was noted in other antimicrobials as well and concluded that disc diffusion is just a screening method, while MICs must be used especially in case of MDR and XDR bacterial isolates.

**Table-II:** Susceptibility data for *P. aeruginosa* isolates MICs (E-test) method.

Antimicrobial Agents	Breakpoints for resistance (µg/dL)	XDR isolates (n=8)		

		S (%)	I (%)	R (%)
Imipenem	≥8	00	25	75
Tigecycline	≥8	25	12.5	62.5
Amikacin	≥64	12.5	00	87.5
Ceftazidime	≥32	00	00	100
Ciprofloxacin	≥4	00	00	100

**Table-III:** Susceptibility data for *P. aeruginosa* isolates MICs broth microdilution method.

	Breakpoints for resistance (µg/mL)	XDR isolates (n=8)		

		S (%)	I (%)	R (%)
Colistin	≥8	62.5	00	37.5
Polymyxin-B	≥8	50	25	25

Out of (n=23) *P. aeruginosa* only MDR and XDR isolates (resistant to 3^rd^ generation cephalosporin), were screened phenotypically for ESBL production. Among which (9/11, 81.8%) were screened as ESBL positive (Table-IV). All the carbapenem-resistant (XDR) isolates of *P. aeruginosa* were tested for carbapenem inactivation activity. The sensitivity of the test in *P. aeruginosa* was 75% (Table-IV).

**Table-IV:** No and percentage of ESBL producing & carbapenemase inactivation by mCIM in MDR & XDR isolates of *P. aeruginosa*.

Organism	ESBL Positive (DDST)	ESBL Negative	mCIM Positive	mCIM Negative
*P. aeruginosa*	9/11 (81.8%)	2/11 (18.1%)	(6/8) 75%	(2/8 )25%

We have detected ESBL and carbapenemase genes in the extensively resistant clinical isolates of *P. aeruginosa* (n=8), among which bla_IMP_ genes were (n=7/8) 87.5%, bla_NDM-1_ (n=5/8) 62.5%), and bla_SHV_ were (n=4/8) 50%. The co-existence of multiple antibiotic-resistant genes bla_SHV_, bla_IMP_, and bla_NDM_ was found in (n=2/8) 25% isolates.

## DISCUSSION

Antibiotic resistance causes a major public health threat across the globe and is increasing gradually, mostly in developing countries. It is the irrational use of antibiotics that has given rise to resistance against a range of antimicrobials. Infections caused by bacterial superbugs are challenging and difficult to treat. This challenge cuts across the developed and developing countries of the world because there is a narrow therapeutic option left, as very few antibacterial agents such as carbapenems, tigecycline, and colistin are available.^19^

All the clinical isolates of *P. aeruginosa* included in the present study were from hospital settings. This was in accordance with the results reported by,^20^ in which it was documented that *P. aeruginosa* is medically important because it causes nosocomial infections throughout the world. The movement of genes and other sequences of the DNA plays an active role in the spread of drug resistance.

In the present study (n=350) clinical isolates were included for the detection of multi and extensive, drug-resistant *P. aeruginosa*. Among the gram-negative bacilli (n=157) *P. aeruginosa* were (n=23) 14.6 %. The persistent spread of ESBLs mediated resistance has intensely increased in both hospitals and the community. It was observed in our isolates that ESBL producers were (n=9/11) 81.8%, which were higher than the results reported by^21^ (69.5%) and almost doubled than the earlier study reported by,^22^ in which (46.8%) of the isolates were ESBL producers phenotypically. This increasing percentage is very alarming in short period. Similarly, MBL (carbapenemases) (6/8) 75% were detected in the present study. It was observed that among the isolates of *P. aeruginosa* (n=8) tested by the mCIM, one isolate was detected as false negative. The false-negative result missed by mCIM was linked to blaIMP (1/8) carbapenemases. The missed isolate was confirmed as PCR positive for the relevant gene. In contrary, lower incidence of MBL (carbapenemases) producers was documented by,^23^ in Egypt (64.8%), and^24^, 37.6%. This rapid increase is again alarming in the region.

Our results showed variable prevalence for MBL (carbapenemase) genes, among which the most prevalent genotype was, bla_IMP_ (n=7/8) 87.5% followed by bla_NDM-1_ (n=5/8) 62.5%, and for ESBL genes like bla_SHV_ (n=4/8) 50%, this was higher than the results reported from Kampala, Uganda, at Mulago hospital in which the frequency of carbapenemase genes like bla_IMP_ were (9/25) 36%, bla_VIM_ (8/25) 32%, bla_NDM-1_ (1/25) 4% in carbapenem-resistant *P. aeruginosa*.^25^

The co-existence of multiple antibiotic-resistant genes bla_IMP_, bla_NDM-1_ and bla_SHV_, in the present study was found in (n=2/8) 25% isolates, in contrary, our results showed a high percentage (25%) of combination genes than reported by^23^, (8.5%) in the combination of five genes, and (2.1%) in a combination of three genes like, bla_NDM_, bla_IMP_, and bla_TEM,_ but bla_TEM_ was reported in combination instead of bla_SHV_. Similarly, combination of two genes blaCTX-M/blaSHV is reported in the isolates of *Klebsiella pneumonia* from tertiary care hospitals in Lahore, Pakistan.^26^

This higher prevalence is the indication of extensive drug resistance against multiple antimicrobials. The spread dynamics of antibiotic resistance is more complex and need more studies in-depth together with whole genome sequencing or multilocus sequence typing to investigate epidemiological evidence of transmission of ESBL and MBL (carbapenemases) in the region. Moreover, the present study is clinically more significant, as it will create awareness among the physicians and common people and help in careful selection of antimicrobials. Furthermore, infection control measures and over the counter usage of antimicrobials must be prevented by health authorities following implementation of strict rules and regulations.

### Limitations:

Although it was a multi-setting study from different tertiary care hospitals of Quetta city that treat the patients even from peripheries of the province, the results cannot be generalized to the patients of the other provinces. Further studies are required to limit the increasing and alarming antimicrobial resistance.

## CONCLUSION

The MBL and ESBL producers are an emerging threat in the region. Resistance of the commonly used antibiotics against carbapenem-resistant bacterial species has been developed. It is of great importance to determine the resistance mechanism and eliminate its root cause. Culture and sensitivity must be considered as an essential elements before going towards antibiotic therapy. The spread of ESBL and MBL, antimicrobial resistance, and the lack of new antimicrobials particularly against gram-negative bacteria is quite alarming globally.

### Authors’ Contribution:

**MD and MA:** Conceived the presented idea, planning of the work and designed the basis of the manuscript. Contributed to PCR and lab work, prepared the manuscript and provided data.

**SRL:** Analyzed the data, and developed the theoretical part.

**MA:** Analyzed and interpreted the data.

**MD:** Is responsible for the accuracy and integrity of the work.

All authors contributed equally to this work and they provided critical feedback, helped shape the research and approved the final version of the manuscript for publication.
